# Short-term follow-up of antibiotic-loaded calcium sulfate in treating chronic periprosthetic joint infection during two-stage revision

**DOI:** 10.3389/fbioe.2025.1352895

**Published:** 2025-01-30

**Authors:** Xiao Sun, Jun Tan, Lijuan Zhan, Mingkui Sheng, Zhongxin Tang, Lingxiao Wu, Jianzhong Xu, Haijun Ma

**Affiliations:** ^1^ Department of Mini-Invasive Spinal Surgery, The Third People’s Hospital of Henan Province, Zhengzhou, Henan, China; ^2^ Department of Neurology, People’s Hospital of Zhengzhou, Zhengzhou, Henan, China; ^3^ Department of Orthopedic Surgery, The First Affiliated Hospital of Zhengzhou University, Zhengzhou, Henan, China

**Keywords:** calcium sulfate, antibiotic, periprosthetic joint infection, two-stage revision, arthroplasty

## Abstract

**Background:**

Periprosthetic joint infection (PJI) is a significant and challenging complication following total knee arthroplasty (TKA). This study aimed to evaluate the efficacy and safety of treating chronic knee PJI with and without antibiotic-loaded calcium sulfate during two-stage revision surgery.

**Methods:**

This retrospective study analyzed 94 patients with TKA infections who underwent two-stage revision between May 2017 and January 2022 at the First Affiliated Hospital of Zhengzhou University. Key outcomes assessed included infection recurrence rates, postoperative range of motion (ROM), Knee Society Score (KSS), Hospital for Special Surgery (HSS) scores, hematological parameters, and complication rates during the follow-up period.

**Results:**

The demographic characteristics of the two groups showed no significant differences. The infection control rate was significantly higher in the calcium sulfate group (95.7%) compared to the matched control group (80.9%) (*P* < 0.05). Both groups demonstrated statistically significant improvements in ROM, HSS, and KSS scores compared to preoperative values (*P* < 0.05). However, intergroup differences in these outcomes were not statistically significant (*P* > 0.05). Additionally, there was no significant difference in postoperative complication rates between the two groups.

**Conclusion:**

The use of antibiotic-loaded calcium sulfate in two-stage revision surgery for chronic knee PJI ensures sustained local antibiotic release at high concentrations, leading to rapid reduction in inflammatory markers, effective infection control, and a low complication rate. This approach is a safe and effective treatment for chronic knee PJI.

## 1 Introduction

Periprosthetic Joint Infection (PJI) is a serious complication of Total Knee Arthroplasty (TKA), affecting 1%–2% of patients and posing a substantial burden on individuals and the global healthcare system (Nelson et al., 2023; Patel, 2023). The primary objectives of PJI management are eradicating infection, relieving pain, and restoring joint function (Rajput et al., 2022). Treatment choice depends on the Tsukayama classification and the patient’s clinical condition. Key treatment options include antibiotic therapy, debridement with prosthesis retention, one- or two-stage revision after debridement, knee fusion, and amputation (Fiore et al., 2022). Two-stage revision surgery, involving temporary spacer implantation and concurrent antibiotic therapy, is a widely adopted approach for advanced chronic periprosthetic infections, achieving infection control rates of 85%–95% (Charette and Melnic, 2018; Craig et al., 2022). The primary strategy of PJI treatment is the combination of local and systemic antibiotic application with surgical intervention. However, systemic antibiotic therapy is often limited by poor penetration and insufficient blood supply to the infected area (Wiesli et al., 2022). Prolonged high-dose antibiotic use can promote the emergence and dissemination of antibiotic-resistant genes in gut flora, escalating bacterial resistance and triggering severe systemic adverse effects (Barrett et al., 2014; Francino, 2015; Zhang and Chen, 2019).

In contrast, local antibiotic delivery systems (DDS) achieve high antibiotic concentrations at the infection site, effectively reducing or preventing adverse reactions linked to systemic therapy. Therefore, selecting an appropriate antibiotic carrier during two-stage revision is vital (Risitano et al., 2018). Recently, biodegradable calcium sulfate has gained popularity in managing PJI during two-stage revision surgeries. It can fully release antibiotics during degradation, creating an environment unfavorable for bacterial colonization (Moore et al., 2021; Levack et al., 2022). These characteristics make calcium sulfate an optimal carrier for localized antibiotic delivery.

This retrospective study aimed to share our experience with two-stage revision procedures using antibiotic-loaded calcium sulfate beads, emphasizing their efficacy in eradicating infection and enhancing joint mobility.

## 2 Materials and methods

### 2.1 Patient selection

This study was approved by the Medical Ethics Committee of the First Affiliated Hospital of Zhengzhou University (No: 2020-KY-495). Informed consent was obtained from all patients, and the study adhered to the principles of the Declaration of Helsinki. This study included patients with infected TKA treated at our center between May 2017 and January 2022 who received antibiotic-loaded calcium sulfate beads during two-stage revision (calcium sulfate group). These patients were compared with a matched cohort who underwent two-stage revision without the use of antibiotic-loaded calcium sulfate beads (matched control group).

Infection was diagnosed according to the Musculoskeletal Infection Society (MSIS) criteria for PJI (Parvizi et al., 2018). All patients were categorized according to the Tsukayama classification (Tsukayama et al., 2003), which divides PJI into four categories according to the time elapsed since prosthesis implantation (Supplementary Table S1). The inclusion criteria were: (1) primary TKA infection classified as Tsukayama type IV; (2) treatment with two-stage revision surgery, with or without antibiotic-loaded calcium sulfate; (3) no prior TKA infection; and (4) availability of complete medical records. The exclusion criteria were: (1) a history of multiple prior knee revision surgeries; and (2) comorbidities associated with severe immune deficiency, such as human immunodeficiency virus (HIV) infection, systemic lupus erythematosus (SLE), malignant tumors, or nephrotic syndrome.

### 2.2 Preparation of calcium sulfate beads

Antibiotic beads were prepared by combining calcium sulfate with antibiotics selected based on preoperative synovial fluid culture results (Abosala and Ali, 2020; Tao et al., 2022; Iorio et al., 2023). For Gram-positive bacterial infections, 2 g of vancomycin was combined with 10 cc of calcium sulfate powder (Stimulan, Biocomposites Inc., Wilmington, NC, United States). For Gram-negative bacterial infections, 1 g of vancomycin and 1.5 g of meropenem were combined with 10 cc of calcium sulfate powder. For fungal infections, 1 g of vancomycin and 50 mg of amphotericin B were combined with 10 cc of calcium sulfate powder. For *Mycobacterium tuberculosis* infections, 3 g of streptomycin and 1 g of vancomycin were combined with 10 cc of calcium sulfate powder. For culture-negative cases, 1.5 g of vancomycin and 1.5 g of meropenem were routinely combined with 10 cc of calcium sulfate powder.

**FIGURE 1:**
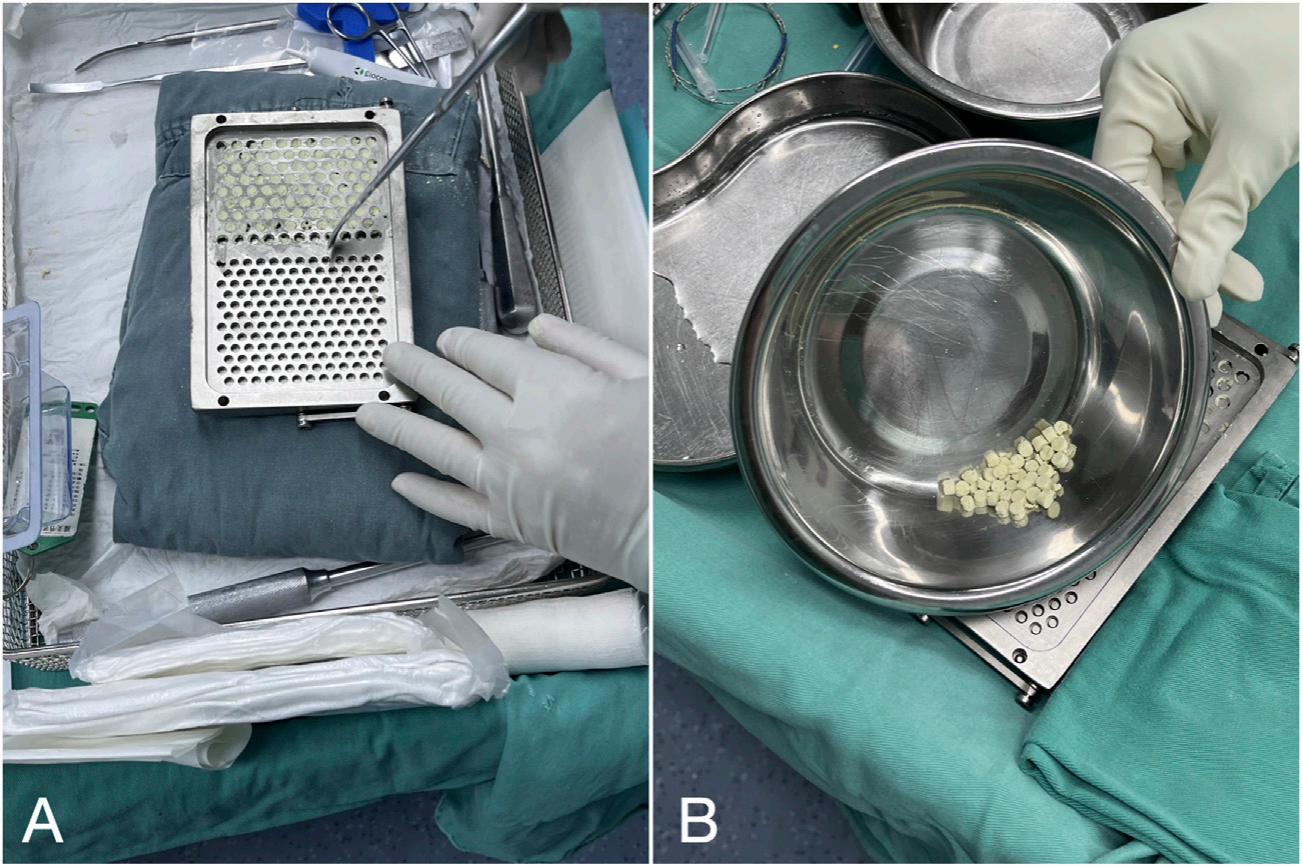
Preparation of antibiotic-loaded calcium sulfate beads: **(A)** Compress calcium sulfate paste into a 4.8 mm diameter mold. **(B)** Remove the solidified beads from the curing mold.

A smooth paste was first prepared within approximately 30 s and transferred into a mold containing cavities with a diameter of 4.8 mm. The paste was then allowed to solidify for 15–30 min before the cured beads were removed from the mold (Figure 1).

### 2.3 Surgical technique

All patients underwent two-stage revision surgeries under epidural or combined spinal-epidural anesthesia. The procedures were performed by a surgical team consisting of a chief orthopedic surgeon and three senior orthopedic surgeons in an operating room with vertical laminar airflow.

#### 2.3.1 First stage procedure

**FIGURE 2:**
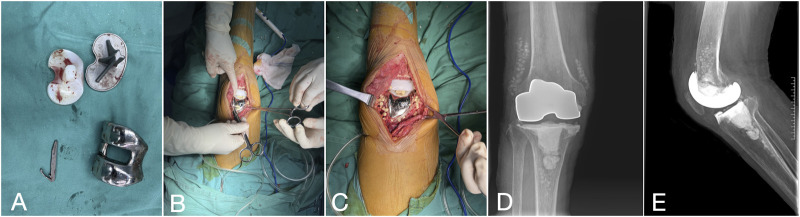
The first-stage procedure. **(A)** Extraction of the infected prosthesis **(B, C)** Involved implanting a modified articulating spacer and positioning antibiotic-loaded calcium sulfate pellets around the prosthesis. **(D, E)** Two weeks post-surgery, the right knee joint’s positive and lateral radiographs display a properly positioned spacer with gradual dissolution of the antibiotic-loaded calcium sulfate beads.

After anesthesia, the knee joint was accessed through the original TKA incision using a medial parapatellar approach. The infected prosthesis and bone cement were removed, followed by meticulous debridement. Joint fluid was aspirated intraoperatively for microbial culture and antibiotic sensitivity testing, and three to five samples of inflammatory tissue were collected for culture. The joint was sequentially irrigated with 3% hydrogen peroxide and 0.9% saline, thoroughly washed, and soaked in 0.5% iodophor to eliminate potential dead space. A pulsatile irrigation device with 0.9% saline was then used for stepwise irrigation from superficial to deep layers. The removed prosthesis underwent ultrasonic lysis (40 Hz, 3 min), and the lysate was centrifuged (4,000 rpm, 15 min) to obtain the final microbial culture sample. To address the gap caused by infected bone removal, a new femoral component and a thicker polyethylene insert were implanted. A modified articulating spacer was used to maintain joint mobility, and a bone cement spacer was placed in the tibia. In the calcium sulfate group, antibiotic-loaded calcium sulfate beads were positioned in the supra-patellar pouch and around the tibia and femur, while in the matched control group, local antibiotics were applied. Following the completion of all procedures, joint mobility and spacer stability were reassessed, and the surgical incisions were routinely closed (Figure 2).

#### 2.3.2 Management of the interval period

Antibiotic selection was guided by microbial culture and susceptibility testing. Following the Infectious Diseases Society of America (IDSA) clinical practice guidelines, sensitive antibiotics were administered intravenously for 2–6 weeks after the first stage procedure. In culture-negative cases, an empirical regimen of vancomycin combined with meropenem was prescribed. Upon discharge, oral rifampicin with levofloxacin or ciprofloxacin was recommended for continued anti-infective therapy. For patients unable to tolerate quinolones, alternatives such as cotrimoxazole, minocycline, cephalexin, or penicillin were suggested.

Intravenous doses of 1.5 g tranexamic acid were administered intraoperatively and at 3 and 6 h postoperatively. Subcutaneous injections of 2.5 mg fondaparinux sodium were initiated 8 h post-surgery and continued once daily for 5–9 days. Postoperative knee wounds were initially dressed with a compression bandage, removed after 6 h to alleviate periarticular pressure, and replaced with a standard adhesive bandage on day 4. Progression to the second stage of surgery was determined after a 6-week antibiotic cessation if infection control was achieved, based on the patient’s condition and preferences in consultation with the physician.

#### 2.3.3 Second stage procedure

**FIGURE 3:**
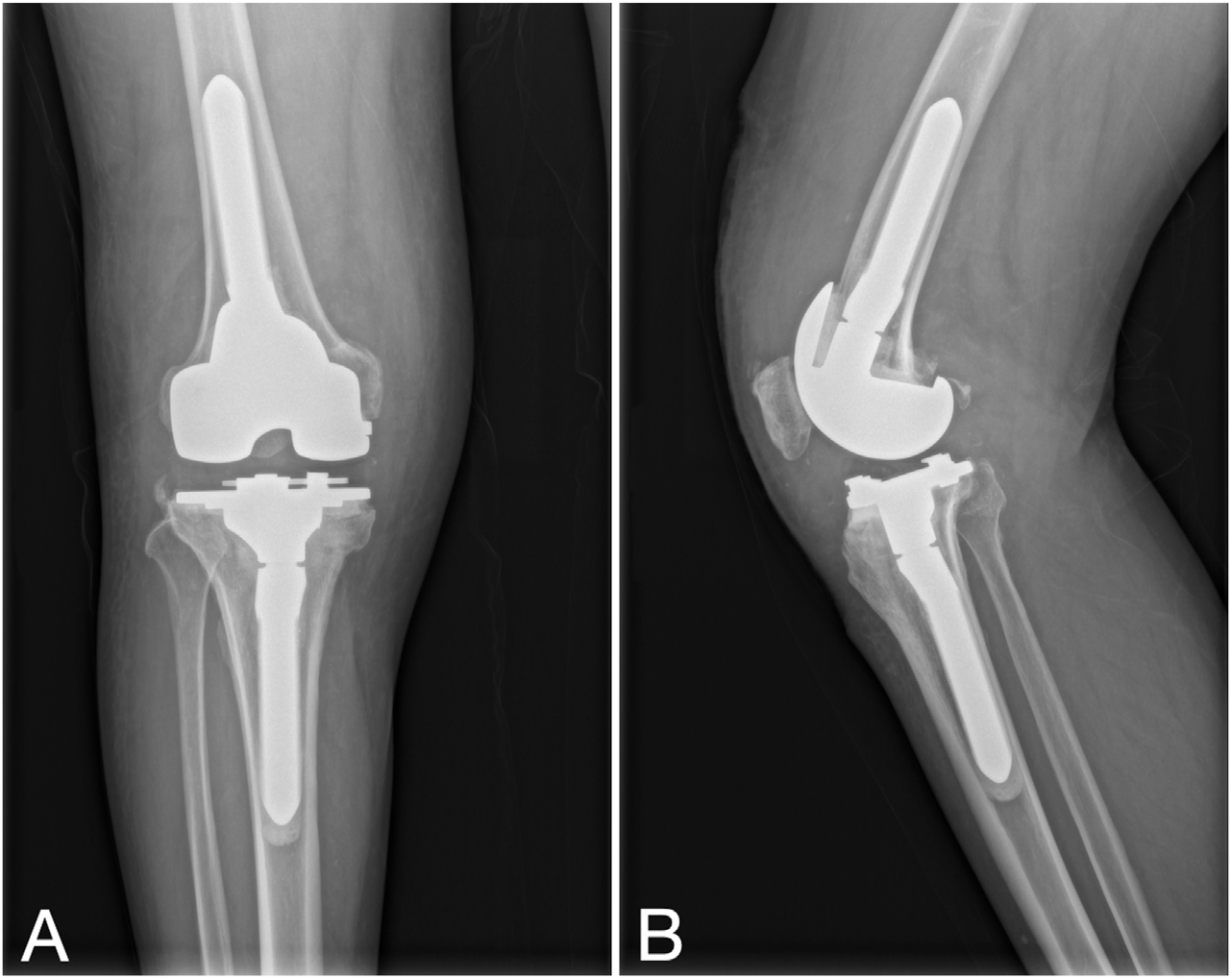
Second-stage procedure with implantation of a new revision prosthesis. **(A, B)** Knee X-rays depict the prosthesis in a satisfactory position.

After anesthesia, the spacer was removed through the original incision, followed by meticulous debridement. The selection of the revision prosthesis was guided by the severity of localized bone defects and the tension of adjacent soft tissues. If the intraoperative frozen section analysis revealed a neutrophil count greater than 5 per high power field (HPF) in all 5 specimens, the revision procedure was aborted, and patients were rescheduled for an additional first-stage procedure. After the second-stage revision, the antibiotic regimen remained consistent with the interval period: intravenous administration of sensitive antibiotics for 2–6 weeks, transitioning to oral antibiotics, with the duration adjusted based on follow-up evaluations (Figure 3).

### 2.4 Definition of infection remission and follow-up

The criteria for infection eradication were based on the Delphi International Multidisciplinary Consensus Criteria, which include: (i) complete resolution of infection with wound healing free of sinus tract or persistent pain; (ii) no need for additional surgical interventions to address infection following prosthesis implantation; and (iii) absence of PJI-related mortality. Clinical and radiological follow-ups were conducted at 1, 3, and 6 months postoperatively, and subsequently on an annual basis. Evaluations encompassed range of motion (ROM), American Knee Society Score (KSS), Hospital for Special Surgery (HSS) score, serologic markers, infection recurrence, and reoperation rates.

### 2.5 Statistical analysis

Statistical analyses were conducted using SPSS 23.0 (IBM Corp., Armonk, NY, United States). Normality tests were applied, and continuous variables were expressed as mean ± standard deviation or median (interquartile range), while categorical variables were presented as number (percentage). An independent samples *t*-test was used for comparisons between the two groups, and a paired *t*-test was used for comparisons between preoperative and postoperative periods. Repeated measures ANOVA was used for comparisons between different time points. The chi-square test and Fisher’s exact test were used for categorical data. Differences were considered statistically significant at *P* < 0.05.

## 3 Results

A total of 103 patients were initially enrolled in the study. Six were excluded due to incomplete data, and three opted out of the second-stage procedure, citing perceived improvement in knee function during the interval period. The final analysis included 47 patients (21 males, 26 females) in the calcium sulfate group, with a median age of 65.6 ± 10.5 years, and 47 patients (19 males, 28 females) in the matched control group, with a median age of 67.1 ± 12.3 years. Twenty patients had undergone primary TKA at our institution, while the remaining 74 were referred from other institutions with a confirmed diagnosis of PJI. No significant differences in demographic characteristics were observed between the two groups (*P* > 0.05, Table 1).

**TABLE 1:** Demographic characteristics.

Categories	Calcium sulfate group	Matched control group	*P*
Age (year)	65.6 ± 10.5	67.1 ± 12.3	0.107
Sex (M/F, n)	21/26	19/28	0.677
BMI (kg/m^2^, mean ± SD)	26.2 ± 3.7	25.8 ± 3.1	0.128
Drinking history (n)	8	5	0.370
Smoking history (n)	13	7	0.131
Site of infection (n)			0.680
Right	23	25	
Left	24	22	
Comorbidities (n)			
Hypertension	8	4	0.216
Diabetes	5	2	0.231
Hyperlipidemia	7	3	0.181

### 3.1 Microbial culture results

Intraoperative specimen cultures yielded positive results in 18 patients (38.3%) in the calcium sulfate group and 16 patients (34.0%) in the matched control group. The difference in positive microbial culture rates between the two groups was not statistically significant (*P* > 0.05). Detailed preoperative and intraoperative culture results are provided in Supplementary Table S2.

### 3.2 Blood test results

In the calcium sulfate group, the mean values for white blood cell (WBC) count, erythrocyte sedimentation rate (ESR), and C-reactive protein (CRP) level before the first and second stage procedure were 13.67 × 10^9^/L and 6.44 × 10^9^/L; 49.71 and 18.79 mm/h; 45.13 and 7.82 mg/L, respectively (*P* < 0.05). In the matched control group, the mean values for WBC count, ESR, and CRP levels before the first and second stage procedure were 15.35 × 10^9^/L and 7.82 × 10^9^/L; 54.23 mm/h and 19.58 mm/h; 44.81 and 9.63 mg/L, respectively (*P* < 0.05). The difference between the two groups was not statistically significant (*P* > 0.05, Supplementary Table S3).

### 3.3 Clinical evaluation results

Postoperatively, both groups demonstrated significant improvements in mean ROM, KSS, and HSS compared to preoperative values (*P* < 0.05). However, intergroup comparisons of ROM, KSS, and HSS at all time points revealed no statistically significant differences (*P* > 0.05, Table 2).

**TABLE 2:** Outcome measures.

Assessment	Calcium sulfate group	Matched control group	*P*
ROM			
First stage preoperative	73.17 ± 16.08	72.61 ± 5.98	0.852
Second stage preoperative	94.69 ± 13.64*	90.78 ± 9.46*	0.723
Terminal follow-up	105.57 ± 13.42*	104.87 ± 9.43*	0.812
KSS			
First stage preoperative	36.14 ± 6.07	33.96 ± 4.96	0.532
Second stage preoperative	75.29 ± 8.41*	75.43 ± 9.16*	0.901
Terminal follow-up	86.14 ± 5.68*	84.04 ± 6.16*	0.827
HSS			
First stage preoperative	59.46 ± 14.77	57.17 ± 11.75	0.539
Second stage preoperative	75.14 ± 11.69*	74.74 ± 9.94*	0.893
Terminal follow-up	84.29 ± 10.15*	83.74 ± 7.65*	0.617

ROM, range of motion; HSS, hospital for special surgery score; KSS, american knee society score.

*Represents a significant difference to the first stage preoperative value.

Infection eradication was achieved in 83 of 94 patients (88.3%). The calcium sulfate group showed a significantly higher success rate (95.7%) compared to the matched control group (80.9%) (*P* < 0.05).

### 3.4 Complications

In the calcium sulfate group, two patients developed asymptomatic heterotopic ossification (HO) in the suprapatellar soft tissue of the distal femur, which was easily removed during the second-stage surgery. Additionally, four patients experienced deep vein thrombosis in small veins distal to the knee joint (one in the calcium sulfate group and three in the matched control group). No significant difference in complication rates was observed between the two groups (*P* > 0.05).

## 4 Discussion

Treating periprosthetic infections after TKA remains a key area of clinical research. For advanced chronic infections or cases lacking preoperative etiological evidence, two-stage revision surgery with temporary spacer implantation and antibiotic therapy is the widely accepted approach (Chang et al., 2019). This approach provides benefits including early joint mobilization, shorter hospital stays, and reduced postoperative infection recurrence rates (Craig et al., 2022). The articulating spacer replicates the knee joint’s anatomical structure, aiding in the prevention of joint contracture and the maintenance of range of motion (Vasarhelyi et al., 2022). Furthermore, Skwara et al. found no significant difference in infection control outcomes between articulating and static spacers (Skwara et al., 2016). Along with radical surgical debridement, effective treatments may involve long-term suppressive antibiotic therapy and local antibiotic delivery via devices made from biodegradable or non-biodegradable materials. Systemic antibiotic therapy often fails to achieve sufficient antibiotic concentrations at the infection site due to poor local blood supply.

Local antibiotic delivery systems, which form a DDS, are widely utilized as they achieve high local drug concentrations while minimizing systemic exposure, thereby reducing the risk of severe side effects (Hasegawa et al., 2021). Studies have shown that effective antibiotic concentrations in infected bone areas persist in surrounding tissues for up to 6 weeks after the topical application of antibiotic delivery devices. Biodegradable delivery systems (such as calcium sulfate) and non-biodegradable delivery systems (such as bone cement) are common carriers for local DDS, both having a substantial history of clinical application. Since Buchholz et al.'s pioneering attempt in 1970 to use antibiotic-loaded bone cement for managing joint infections, this method has gained widespread recognition as an effective adjunctive therapy for orthopedic infections. Antibiotic-loaded bone cements are frequently used as spacers, serving to elevate local antibiotic concentrations, mitigate soft-tissue contracture, allow partial weight-bearing, and alleviate movement restrictions (Roof et al., 2021). Additionally, these cements can fill intra-articular defects left by removed components, potentially aiding in the recovery of knee joint function.

Initially, bone cement releases antibiotics rapidly, but over time, its release kinetics become sub-therapeutic, resulting in decreasing levels of eluted antibiotics. Studies have reported a reduction to 10% of the initial concentration within 24 h post-implantation (McKee et al., 2010). If the antibiotics released from bone cement fall below the minimum inhibitory concentration, pathogens may colonize the surface of cement spacers, leading to biofilm formation. This bacterial biofilm could impede antibiotic penetration, potentially exacerbating chronic infection and its recurrence (Howlin et al., 2015).

Biodegradable calcium sulfate beads provide an alternative method for localized antibiotic delivery in infected areas. The application of calcium sulfate for osteomyelitis treatment dates back to 1892 (Laycock et al., 2018). This delivery device features a porous structure that allows effective local antibiotic delivery and complete *in vivo* absorption. Antibiotics released from calcium sulfate beads exhibit zero-order kinetics, which effectively mitigates biofilm formation and potentially inhibits bacterial colonization. Their exceptional elution properties maintain high local antibiotic concentrations continuously for 4–6 weeks. This enables antibiotics to reach the minimal bactericidal concentration within biofilms at infected sites, ensuring low systemic and local toxicity. This is crucial for effectively controlling infection during the interval period (Cooper et al., 2016; Smolle et al., 2023). The study by Howlin et al. demonstrated that antibiotic-loaded calcium sulfate beads can eradicate 10^6 CFU/mL planktonic cultures, inhibit bacterial colonization, and significantly reduce biofilm formation within days (Howlin et al., 2015). The synergistic effect between antibiotics and calcium sulfate enhances porosity, facilitating increased antibiotic release and broadening the antibacterial spectrum. Studies have shown that the combination of multiple antibiotics with calcium sulfate increases and prolongs antibiotic release compared to a single antibiotic (Levack et al., 2022).

This study demonstrated effective infection control in both groups following standardized treatment, with the calcium sulfate group exhibiting a higher infection control rate than the matched control group (95.7% vs. 80.9%). A review by Pangaud et al. (2019), which included 18 studies from 2000 to 2018, reported a mean infection control rate of 85% for chronic PJI of the knee treated with two-stage revision. Similarly, a multicenter retrospective study by Kildow et al. (Kildow et al., 2022) on two-stage revision for chronic knee PJI found an overall infection control rate of 88.98%. Kallala and Haddad (2015) reported a 93.3% infection control rate in PJI revision using antibiotic-loaded calcium sulfate. Lum and Pereira (2018) also reported a high infection control rate, achieving 100% in a study of 14 patients with PJI using antibiotic-loaded calcium sulfate. In this study, the calcium sulfate group achieved an infection control rate superior to that in earlier studies, while remaining consistent with recent findings. This superior infection control rate can be attributed to several key factors, including thorough debridement of infected tissues, excellent intraoperative visualization, an expanded scope of debridement, and the use of local antibiotic carriers. For patients with periprosthetic joint infection, bacteria are primarily located at the prosthesis-tissue interface, where they secrete a polysaccharide-protein complex and adhere to form bacterial biofilms. These biofilms effectively isolate bacteria from antibiotics, making their eradication challenging. To address this, our approach in the first-stage procedure involved the implantation of a new femoral prosthesis rather than reimplanting the original prosthesis sterilized by high-pressure techniques, as traditionally performed. This strategy, combined with the effective local antibiotic release provided by calcium sulfate beads, was pivotal in achieving the high infection control rate observed in our study.

The two patients in the calcium sulfate group who failed infection control were found to have methicillin-resistant *Staphylococcus aureus* (MRSA) in both pre- and post-treatment cultures. The risk of reinfection following revision surgery for MRSA-induced PJI was 20.5 times higher compared to PJI caused by sensitive or unidentified bacteria, with the reinfection rate increasing from 19% to 52% (Vasso et al., 2016). Lim et al. (2009) found that in a study comparing two-stage revision outcomes, 33% of patients with PJI caused by resistant bacteria experienced infection recurrence, while no recurrence was observed in those with non-resistant infections. Given the high virulence and recurrence risk associated with pathogens such as fungi, drug-resistant bacteria, and coagulase-negative staphylococci, we recommend thorough debridement, strict antimicrobial strategies, and comprehensive patient education to enhance treatment compliance.

In a retrospective analysis of the use of antibiotic-loaded calcium sulfate beads in infection-related revision arthroplasty, Kallala et al. reported three instances of hypercalcemia among 15 patients, with an average follow-up period of 16 months (Kallala and Haddad, 2015). Although hypercalcemia is considered a potential complication following the use of calcium sulfate, it was not observed in the present study. However, five cases of asymptomatic HO occurred after the first-stage surgery. While the exact reasons for HO remain unclear, it is plausible that the excessive aggregation of calcium sulfate beads may elevate local calcium concentrations, thereby increasing the risk of HO (Kallala et al., 2018).

This retrospective analysis highlights the importance of thorough removal of infected tissue, optimal intraoperative visualization, a broader debridement area, and careful selection of local antibiotic delivery systems to improve surgical success rates. In periprosthetic infections, bacteria primarily colonize the active interface of the prosthesis, secreting polysaccharide-protein complexes to form protective biofilms. As biofilms protect bacteria from antibiotics, replacing the femoral prosthesis is crucial for ensuring effective debridement.

## 5 Limitations of this study

This study has several limitations, including its retrospective design and the lack of prior sample size calculation. The retrospective nature of the study prevented the assessment of additional potential risk factors for recurrence or persistent infection, such as alcoholism, prior intra-articular corticosteroid injections, cutaneous ulcers, and diabetes. Additionally, the study had relatively low detection rates for bacterial cultures and histopathological examinations, which may have increased the likelihood of false-negative results. Future research should explore alternative detection methods, such as metagenomic sequencing, to improve detection rates, especially in false-negative cases. Therefore, a large-scale randomized controlled trial is necessary to thoroughly evaluate the effectiveness of antibiotic-loaded calcium sulfate beads in treating TKA infections during two-stage revision.

## 6 Conclusion

The use of antibiotic-loaded calcium sulfate in two-stage revision surgery for chronic knee PJI ensures sustained local antibiotic release at high concentrations, leading to rapid reduction in inflammatory markers, effective infection control, and a low complication rate. This approach is a safe and effective treatment for chronic knee PJI.

## Data Availability

The original contributions presented in the study are included in the article/Supplementary Material, further inquiries can be directed to the corresponding author.
