# Supplementary material for: Short-term follow-up of antibiotic-loaded calcium sulfate in treating chronic periprosthetic joint infection during two-stage revision
Source: Front Bioeng Biotechnol. 2025 Jan 30;13:1352895. doi: 10.3389/fbioe.2025.1352895 (PMC11821931; doi:10.3389/fbioe.2025.1352895)
Supplement: Supplementary file 1 [file Table1.docx]

**Supplementary Table 1.** Tsukayama classification of infection.

| **Categories** |  |
| --- | --- |
| I. | Positive intraoperative culture |
| II. | Early postoperative infection: A. Superficial; B. Deep |
| III. | Acute hematogenous |
| IV. | Late chronic |

**Supplementary Table 2.** Microbiology before and after surgery.

| Types of bacteria | Calcium sulfate group | |  | Matched control group | |
| --- | --- | --- | --- | --- | --- |
|  | Pre-operation | Intra-operation |  | Pre-operation | Intra-operation |
| Staphylococcus aureus | 4 | 6 |  | 2 | 5 |
| Staphylococcus epidermidis | 2 | 3 |  | 1 | 2 |
| Staphylococcus Hemolyticus | - | 2 |  | 1 | 2 |
| Enterococcus faecalis | 1 | 2 |  | 2 | 3 |
| Escherichia coli | 1 | 2 |  | - | 1 |
| Candida parapsilosis | - | 1 |  | 1 | 1 |
| Candida albicans | - | 1 |  | - | 1 |
| Candida glabrata | - | 1 |  | 1 | 1 |
| Total | 8 | 18 |  | 8 | 16 |

*P*>0.05

**Supplementary Table 3.** Blood test results between the two groups.

| Parameter | Calcium sulfate group | Matched control group | *P* |
| --- | --- | --- | --- |
| WBC (×10^9^/L) |  |  |  |
| First stage preoperative | 13.67±4.15 | 15.35±5.09 | 0.902 |
| Second stage preoperative | 6.44±3.28* | 7.82±3.63* | 0.871 |
| ESR (mm/h) |  |  |  |
| First stage preoperative | 49.71±11.84 | 54.23±14.37 | 0.793 |
| Second stage preoperative | 18.79±5.33* | 19.58±7.12* | 0.928 |
| CRP (mg/L) |  |  |  |
| First stage preoperative | 45.13±11.59 | 44.81±12.57 | 0.921 |
| Second stage preoperative | 7.82±2.14* | 9.63±2.86* | 0.916 |

WBC: white blood cell count; ESR: erythrocyte sedimentation rate; CRP: C-reactive protein. *represents a significant difference to the first stage preoperative value.
